# Effectiveness of a diet and physical activity promotion strategy on the prevention of obesity in Mexican school children

**DOI:** 10.1186/1471-2458-12-152

**Published:** 2012-03-01

**Authors:** Teresa Shamah Levy, Carmen Morales Ruán, Claudia Amaya Castellanos, Araceli Salazar Coronel, Alejandra Jiménez Aguilar, Ignacio Méndez Gómez Humarán

**Affiliations:** 1 Nutrition Monitoring, Center for Research in Nutrition and Health, National Institute of Public Health, Morelos, Mexico; 2 Center for Research in Mathematics, AC, Aguascalientes, Mexico; 3 National Institute of Public Health, Av. Universidad # 655, Col. Sta. Ma. Ahuacatitlán, Cuernavaca, Morelos, Mexico 62100 C.P

**Keywords:** Obesity, Scholars, Effectiveness, Mexico, Eating, Physical activity

## Abstract

**Background:**

Overweight and obesity in children in Mexico was among the countries with the highest prevalence's in the world. Mexico currently has few innovative and comprehensive experiences to help curb the growth of this serious public health problem. Therefore, the aim of this study is to assess the effectiveness of a nutrition and physical activity strategy, called "Nutrition on the Go" (*"nutrición en movimiento") *in maintaining the BMI values of school children in the State of Mexico.

**Methods:**

A two-stage cluster trial was carried out. Sixty schools were selected in the State of Mexico, of which 30 were randomly assigned to the intervention group (IG) and 30 to the control group (CG). A total of 1020 fifth grade school children participated. The intervention strategy aimed to decrease the energy content of school breakfasts and include fruits and vegetables, as well as increase physical activity and the consumption of water during the time spent at school. The strategy was implemented over a 6-month period.

**Results:**

The estimated probability (EP) of obesity between baseline and the final stage for the IG decreased 1% (Initial EP = 11.8%, 95%CI 9.0, 15.2, final EP = 10.8, 95%CI 8.4, 13.) For the CG, the probability increased 0.9% (baseline EP = 10.6%; 95%CI 8.1, 13.7; final EP = 11.5, 95%CI 9.0, 14.6). The interaction between the intervention and the stage is the average odd time corrected treatment effect, which is statistically significant (*p *= 0.01) (OR = 0.68, 95%CI 0.52, 091).

This represents the interaction between intervention and stage, which is highly significant (*p *= 0.01) (OR = 0.68; 95%CI 0.52, 091). In addition, girls had a protective effect on obesity (OR = 0.56; 95%CI 0.39, 0.80).

**Conclusions:**

The intervention strategy is effective in maintaining the BMI of school children.

## Background

Obesity has become the global epidemic of the 21^st ^century [1] and a public health problem worldwide, with alarming and widespread growth and both short- and long-term health implications [2]. While some studies have reported that childhood body mass index (BMI) is associated with adult adiposity, the magnitude of this association may depend on the relative fatness of children [3]. Studies have also noted that obesity in childhood was strongly predictive of obesity in early adulthood, nevertheless most obese young adults had a healthy weight as children [4], while overweight or obese children who continued to have these conditions as adults had an increased risk of type 2 diabetes, hypertension, dyslipidemia, and carotid-artery atherosclerosis. The risks of these outcomes for overweight or obese children who later in adulthood were not obese were similar to those for persons who were never obese [5].

According to a report by the member countries of the Organization for Economic Cooperation and Development (OECD), 1 of 2 adults is overweight and 1 of 6 is obese [6]. Mexico has not escaped this situation. In fact, Mexico was among the countries with the most overweight in children and obesity among adults in 2010 [7].

The prevalence of obesity and overweight in Mexico increased between 1999 and 2006, particularly among the adult population (61 to 70%) [8,9]. Additionally, this epidemic has a high rate of growth among children, resulting in a high prevalence of overweight and obesity among school children across the country [5].

Specifically, the State of Mexico, which is the focus of this study, showed a combined prevalence of overweight and obesity of 31.5% [5], which is comparable to that of 2008 (30.4%) [9-11].

As a result of this situation, several research groups have focused their efforts on public health campaigns to prevent obesity in school age children, without yet having clarity as to the approaches that work [12]. They have directed their research to support evidence for interventions to prevent obesity in school children in order to determine the most cost-effective strategies for bringing about results and to generalize those results to other contexts. Various studies about the effects of interventions on school age children using different strategies [13-29] have reported little effect on reducing the prevalence of overweight and obesity, showing only that the effects of the strategies can maintain children's BMI over time.

Mexico currently has few innovative and comprehensive experiences to help curb the growth of this serious public health problem. Therefore, the aim of this study is to evaluate the effectiveness of a diet and physical activity strategy among school age children in the State of Mexico--known as "Nutrition on the Go" http://www.insp.mx/dif -to maintain BMI, as a basis for establishing public health policy.

## Methods

### Study population

A blind cluster-randomized field trial was conducted with fifth grade school children. The sample was representative of the population attending fifth grade elementary schools in the State of Mexico. The study was carried out during the 2010-2011 school year in public elementary schools in the 125 municipalities of the State of Mexico. Subjects were beneficiaries of a school breakfast program in both federal and state educational systems with morning and evening shifts. The baseline assessment was conducted in early November 2010; the strategy was implemented between November 2010 and the first half of May 2011 and the final evaluation was conducted between late May and early June 2011. Two measurements--baseline and final--were conducted during the study. Children with disabilities for whom anthropometric measurements could not be performed were excluded.

### Design and sample

To determine the sample size, age-adjusted BMI was used as the variable of interest, based on the variances and design effects of a previous study with the same population [30]. For BMI, a standard deviation of 2.79 and a design effect of 1.68 were estimated. The minimum detectable difference was considered to be 0.45^a ^BMI units, with a 95% confidence interval (CI) and a power of 80%. A sample size of 1,000 children was calculated [31]. Sixty schools were selected at random, of a total of 2,969 public schools in the State of Mexico that receive school breakfasts. Thirty were randomly assigned to the intervention group (IG) and 30 to the control group (CG). The average group sizes in schools were 40 students. Within each school, 17 fifth grade children were also randomly selected, resulting in a total of 510 children per intervention group in order to have a sufficient sample size at follow-up. The non-response rate expected in this study was ≤ 5%.

### "Nutrition on the go" strategy

The strategy "Nutrition on the Go" consisted of 4 components:

1. A gradual decrease of the energy content of school breakfasts by reducing the fat content in milk, not increasing carbohydrates, decreasing the sugar content of the cereals provided and including fruit.

2. The gradual regulation of food offered within the school, through the technical council of the State of Mexico.

3. Gradual adherence to the physical activity program, according to the requirements of the Ministry of Public Education (SEP, Spanish acronym) [32].

4. Implementation of an educational campaign, called "Healthy Break," for healthy eating and physical activity. The objectives of this program are to promote consuming one fruit and one vegetable, drinking pure water and performing physical activity (organized games and calisthenics) during break. The campaign was developed by identifying the audience and the interests and needs of the school population regarding basic concepts related to nutrition and physical activity, and defining the materials and methods to convey the information.

Based on these factors, an intervention to be implemented in schools was developed according to the perceived needs of the target population and formative research; the resulting intervention was tested on a small scale using an efficacy study.

The educational materials produced for the "Nutrition on the Go" strategy for healthy eating and physical activity included: student booklets and a facilitator's guide; a school guide; a calendar for parents, as well as videos (or printed handouts for schools with no DVD players) and audio spots.

This study used two types of validation: a technical validation which consisted of a review by specialists and approval of the educational content of the material, and a validation at the population level to test, with a representative group from the target population, whether the contents and form of the materials actually work as intended.

The stages of the validation process [33] were: 1) Identify the target population and the content of the campaign; 2) First draft of the campaign and the media guide; 3) Select and train staff to validate the material; 3) Prepare material; 4) Convene a group to validate and validation through a workshop; 5) Analyze results and 6) Change the material according to the validated results. A second validation was performed and, lastly, the process to produce the materials was initiated.

The messages and materials were evaluated by a committee of 38 experts that included academic representatives from the Ministries of Education and Health, NGOs, and food industry representatives, among others. Comments were subsequently requested using a questionnaire and modifications were applied according to the suggestions from the experts.

Eight elementary schools were then selected (4 urban and 4 rural) in which a pilot test with the material was conducted in order to identify its strengths and limitations and make the necessary adjustments. An additional study of the materials was subsequently performed using both a quantitative approach of frequencies and a qualitative study based on the categories analyzed. The criteria explored were: attraction, understanding, identification, acceptance and induction into action, as rated by Ziememdorff [33].

The efficacy study was conducted in 6 schools in the State of Mexico (3 urban and 3 rural) randomly assigned to intervention or control groups. The study's primary audience was the school population between 10 and 12 years of age who were in the fifth and sixth grades of elementary school. Also included were students between first and fourth grade, teachers and parents; a total of 2,762 students participated.

The principal objective was to maintain the BMI after the intervention. The results of this study show that 4 months following the intervention, age-adjusted BMI remained stable (18.6 vs. 18.55). The components of the intervention are described below.

### Stages of the intervention

Stage 1. *Training*. Forty-five promoters were standardized and trained during 3 weeks in the activities that the schools would perform in order to implement the strategy. Anthropometric measurements were obtained by field personnel who were trained and standardized using conventional techniques [34,35]. Agreement and consistency for the process of standardization was obtained using the Cohen's Kappa test [36], with a coefficient of 0.76 (95%CI 0.54, 0.83) for height and for weight of 0.87 (95%CI 0.78, 0.90), which indicated acceptance of the measurements. For the standardization in obtaining information using the questionnaires, field workers underwent repeated practice and were evaluated on an ongoing basis.

Stage 2. *Strategy implementation in schools*. The 60 schools included in the study were visited and baseline evaluations were performed. The intervention was implemented in the 30 schools in the IG for a period of 3 weeks in each school; implementation of the strategy was conducted for 6 months. At the end of the period a baseline assessment both in IG and CG schools was conducted and a final evaluation was carried out 6 months later. Additionally, questionnaires were administered to the students regarding knowledge and self-efficacy in the areas of nutrition and physical activity during both stages.

During implementation in IG schools, a trained and standardized staff member supervised and supported the implementation of the strategy and also evaluated the performance of the activities by monitoring them.

### Intervention description

The intervention took place while school was in session. The ongoing activities in schools in the IG were:

a) Nutrition and physical activity workshops. These were divided into 6 sessions which included participatory recreational activities for children to gain knowledge and skills to properly select healthy foods and promoting physical activity. Teaching resources used included the school guide, facilitator's guide, student booklets, videos and printed material.

The workshop was intended to reinforce and expand knowledge and foster self-assessment, proper food choices and physical activity. The dynamics during each session allowed students to actively participate.

b) Puppet Theatre, based on the theory of peer learning [37]. The fifth grade students participating in the study presented a puppet show to students from first to third grades after they studied the script and rehearsed for the performance. Teaching resources provided to each school included the script of the play, puppets and the backdrop.

These activities were conducted once per week for 4 weeks and the puppet show was presented once per month.

Activities with elementary school teachers included:

c) Two-day workshops in each school to raise awareness about healthy eating and physical activity. The workshops sought to convey to teachers the importance of healthy eating and physical activity through dynamic and playful activities to promote participation. Teaching resources included PowerPoint presentations, games, group dynamics, handouts and descriptive cards.

The workshops were taught by nutritionists and health professionals (nurses and social workers) who were previously trained by nutritionists, psychologists and educators and physical trainers with bachelor degrees.

Activities to change the school environment included:

d) Sale of fruits, vegetables and pure water in the school's store cooperative. A session was held for store personnel to convey information about healthy eating, make suggestions about types of food to sell in schools and recommend the daily sale of vegetables, fruit and pure water. The importance of the responsibility of the cooperative (the food store inside the school) for preserving the health of the school community was addressed. The duration of each session was 1 hour.

e) To promote the consumption of pure water, spots were broadcast using the schools' PA systems, and water bottles were delivered to children and teachers to encourage water consumption. A journal was kept on a sporadic basis to verify that the children were carrying their water bottles with them and that they only contained water.

Activities with the educational community included:

f) Physical activation. Organized activities involving motion were conducted twice per week. Activities performed each day before the start of classes included warm-ups, activation and relaxation. Recommendations to support physical activation were provided through the school guide and a CD with music for established activities. Weekly activation sessions gradually increased from 2 to 5 days.

g) Broadcasting of audio spots on the schools' PA systems. Spots were broadcast 3 times per week during the break. The central messages were aimed at promoting the consumption of fruits, vegetables and pure water during break and to promote physical activity in children, with an average length of 1 min and 15 seconds per spot.

h) Organized games during break (once per week). Active and safe participation of teachers and children was promoted during break. Educational materials were provided for these activities, including posters with suggestions for team games and activities that involved moving during 30 minute breaks. To this end, the schools were provided with balls, ropes and hoops, as well as a guidebook, which also were useful for physical activation sessions and physical education classes.

i) Placement of banners at the entrance of the school. In order to highlight the campaign in the school community, a banner was hung that read, "This school promotes healthy breaks."

Activities with parents included:

j) Delivery of recipe calendars. Calendars were distributed that included healthy recipes for school lunches in order to disseminate information to parents about healthy eating and physical activity.

### Evaluation

A baseline test was conducted in all schools to establish the initial characteristics of intervention and control groups. Information was also obtained related to anthropometrics, socioeconomic level, food, physical activity, self-efficacy and knowledge. The same information was obtained for the final evaluation

### Variables

#### Body composition

#### Body mass index

Weight and height were measured. The promoter, who was previously standardized, asked the children to remove their shoes, wear minimal clothing, and unbraid hair that could interfere with the height measurement. The weight was determined with Tanita electronic scales with an accuracy of 100 g and the height was measured using Dynatop stadiometers with a capacity of 2 m and an accuracy of 1 mm. Weight and height of the children who participated in the survey were measured by a trained and standardized field team [34,35].

BMI (BMI = kg/m^2^) was determined for all students to classify them as adequate BMI, overweight or obese, considering the distribution and cutoff points proposed by the International Obesity Task Force (IOTF) [38].

### Behavioral outcomes

#### Food intake

A Food Frequency Questionnaire (FFQ) was administered to the parents of every child. The FFQ employed was used in the 2006 National Health and Nutrition Survey (ENSANUT-2006) [39].

#### Physical activity

A semi-quantitative questionnaire was used to record the physical activity of students, based on the Youth Activity Questionnaire developed and validated by Hernández et al. 1999 [40].

#### Knowledge

We designed a multiple choice questionnaire with two sections and answers presented graphically, to assess children's knowledge about diet and physical activity. The questionnaire consisted of 13 items, of which 7 corresponded to diet and 6 to physical activity.

#### Self-efficacy

To evaluate the self-efficacy of children with respect to physical activity, a dichotomous scale with 12 items was used, which was designed and validated for school populations by Aedo A. and Avila H. in 2009 [41]. The scale consists of 3 dimensions for self-efficacy: the search for positive alternatives, the ability to face potential barriers and skill or competence related to expectations.

To assess self-efficacy on the topic of healthy eating, certain questionnaires were used as a reference, and the items were adapted to tie them with the physical activity scale, resulting in a dichotomous choice questionnaire consisting of 13 items. The values "1" and "0" were assigned to the dichotomous scale (meaning yes and no, respectively). Both scales have been tested and validated with children of similar ages in the United States [42,43] and were adapted for the characteristics of Mexican children.

The results were obtained from the sum of positive responses (value 1), where the minimum value obtained was "0" and the maximum was "12" or "13," according to the number of items in each scale. The percentage of positive responses was then estimated for each questionnaire.

We previously categorized the level of self-efficacy for each child into 3 categories:

1) Low self-efficacy, rated between 0 to 33.3%, when the child had little confidence in himself or herself in terms of modifying eating or physical activity behavior.

2) Medium self-efficacy, rated between 33.4 to 66.6%, when the child believed he/she could perform various activities (related to his/her nutrition or physical activity) but was not sure about his/her ability to successfully complete them.

3) High self-efficacy, ranked between 66.7 to 100%, when the child was convinced he/she would succeed in performing a certain behavior and was willing to modify his/her actions and behaviors.

To measure self-efficacy, the Eating Self-Efficacy Scale was used, adapted into Spanish [44].

### Adjustment variables

Socioeconomic status was calculated by obtaining the children's age, sex, housing characteristics and possession of goods. A socioeconomic index (SEI) was calculated using the principal components method, with 7 variables, where the first principal component explained 40.2% of the total variance. This in turn was divided into tertiles to obtain socioeconomic levels.

### Ethical aspects of the study

First, a written authorization for the school's participation in the study was requested from school principals and the teachers of the groups. During school meetings, parents were explained the purpose and procedure of the study, the lack of risks, the time needed to administer the questionnaire and the process to measure weight and height, as well as the importance of the participation of the child. They were also informed that the children's participation would be voluntary and that there would be no consequences or limitations to their right to receive school breakfasts if the child withdrew from the study, which he/she could do at any moment. After the explanation, mothers were asked for written informed consent to interview their children.

The same procedure was repeated with the children selected to participate in the study through an agreement letter.

The protocol for this study was submitted at every stage to the ethics, biosafety and research committees at the Mexico National Institute of Public Health.

### Data analysis

For the baseline stage, a descriptive analysis was conducted of frequencies and means, with their respective confidence intervals, in order to observe whether there were differences between intervention and control groups at the beginning of the study.

For the variables related to knowledge, results were compared and analyzed using the average scores obtained by the children from each assessment, based on a scale of 1 to 10 for both topics and the treatment groups.

The following values were used to determine the percentage of students who passed the knowledge questionnaires: > 6 = passed; ≤ 6 = failed (Secretarial Agreement, SEP 2009).

Subsequently, a generalized ordinal logistic regression model was developed in order to observe changes in BMI ordinal categories (normal, overweight and obesity) as a result of the implementation of the strategy (intervention effect TR = 1 as a dummy variable), the stage (baseline or final also using a dummy variable with stage = 1 for final) and its interaction as an estimator of the average effect of the time-corrected effect of treatment (difference-in-differences estimate). The regression variables used were physical activity and the difference-in-differences effect. The following variables were included: physical activity, consumption of food energy, self-efficacy scale and knowledge about eating and physical activity for both cases, adjusted for age, sex and SEI.

As part of the analysis, a validation was conducted in which the possible co-linearity between variables and the corresponding statistical assumptions were verified, as well as the adjustment with the use of the cluster and panel data design structure to correct the standard errors in the estimation of coefficients for models [45]. A significance of p ≤ 0.05 was used for main effects and p ≤ 0.1 for factor interactions. All analyses were conducted using STATA 11, svy and gologit2 free usage routine [46].

## Results

**Figure 1:**
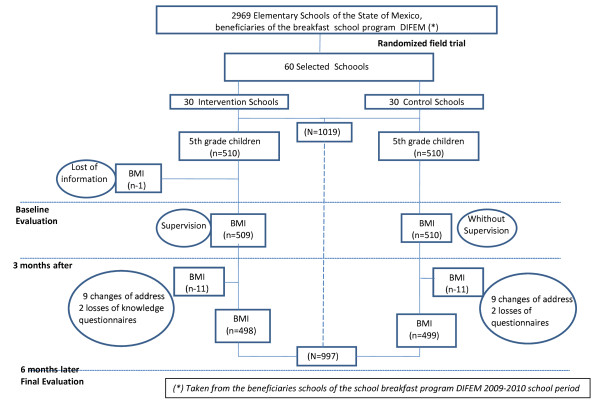
**Selection of study population**.

The loss to follow-up between the baseline and final assessment was only 3.2% and was evenly distributed by treatment group (13 children in the IG and 12 CG). This was less than the maximum non-response rate expected for this study (5%) (Figure 1).

Most of the characteristics for children in the IG and CG at baseline were similar.

While differences were found in consumption and physical activity variables, no significant differences were found in BMI differentiated by sex, or in the prevalence of overweight and obesity. Differences were found between groups in the overall prevalence.

In addition, the distribution by sex, age, socioeconomic status, and the results inform physical activity knowledge and self-efficacy tests showed no statistically significant differences between the two study groups (Table 1).

**Table 1:** Baseline Characteristics of study sample, by treatment group

		Intervention	Control	
		n = 510	n = 509	*p*
**Sex (%)**				
	Females	51.6 (47.2,55.9)	49.7(45.4,54.1)	0.55
	Males	48.4 (44.1,52.8)	50.3 (45.9,54.6)	
**Age (y) (%)**				
	< 10	7.5 (5.2,9.7)	10.5 (7.8,13.1)	0.49
	10	78.6 (75.1,82.2)	75.3 (71.3,78.8)	
	11	10.4 (7.7,13.0)	10.3 (7.6,12.9)	
	12	2.2 (0.9,3.4)	2.8 (1.3,4.2)	
	≥ 13	1.4 (0.4,2.4)	1.2 (0.4,2.7)	
**Socioeconomic Index (SEI) (%)**				
	Low	34.9 (30.8,39.0)	34.1 (30.1, 38.3)	0.12
	Medium	33.7 (29.6, 37.8)	39.3 (35.0, 43.5)	
	High	31.4 (27.3, 35.4)	26.5 (22.7, 30.4)	
**Overweight**				
	Females	20.2 (15.3,25.0)	23.3 (18.0,28.6)	0.39
	Males	19.0 (14.1, 23.9)	25.9 (20.5,31.3)	
**Obesity (%)**				
	Females	11.4 (7.6,15.3)	9.6 (6.0, 13.3)	0.78
	Males	17.4 (12.7,22.2)	15.1 (10.7,19.6)	
**Body Mass Index (BMI) (X)**				
	Females	18.6 (18.1, 19.9)	18.7 (18.3, 19.1)	0.6
	Males	18.8 (18.3,19.2)	18.8 (18.4,19.3)	0.85
**Dietary intake (X)**				
	Energy (Kcal)	1731.0 (1656.5, 1805.5)	1509.2 (1437.6, 1580.8)	0.0001
	Carbohydrates (g)	339.7 (324.0, 355.4)	299.0 (284.2, 313.8)	0.0002
	Lipids (g)	28.9 (27.5, 30.4)	24.3 (23.0, 25.6)	0.0001
**Physical Activity (%)**				
	Non active	21.9 (18.3,25.4)	47 (42.7, 51.4)	0.0001
	Moderately active	14.4 (11.3, 17.4)	14.6 (11.5, 17.6)	
	Active	63.8 (59.6, 68.0)	38.4 (34.2,42.6)	
**Knowledge (X)**				
	Eating	7.5 (7.3, 7.6)	7.3 (7.2, 7.4)	0.04
	Physical Activity	7.2 (7.1, 7.3)	7.2 (7.0, 7.3)	0.4
*Eating (%)*				
	passed > 6	82.1 (78.8, 85.5)	77.2 (73.6, 80.9)	0.05
	failed < 6	17.9 (14.5, 21.2)	22.8 (19.1,26.4)	
*Physical Activity*				
	passed > 6	85.7 (82.6, 88.7)	84.3 (81.1, 87.4)	0.5
	failed < 6	14.3 (11.3, 17.4)	15.7 (12.6,18.9)	
**Self-efficacy (X)**				
	Eating	8.6 (8.5, 8.7)	8.5 (8.4,8.6)	0.2
	Physical Activity	6.8 (6.6, 6.9)	6.7 (6.6,6.9)	0.7
*Eating*				
	High	94.9 (93.0,96.8)	91.9 (89.6, 94.3)	0.1
	Medium	5.1 (3.2, 7.0)	7.9 (5.5, 10.2)	
	Low	**-**	0.2 (0.0, 0.6)	
*Physical Activity*				
	High	43.6 (39.3, 47.9)	42.4 (38.1, 46.7)	0.4
	Medium	53.4 (49.1, 57.8)	53.0 (48.7, 57.4)	
	Low	3.0 (1.5, 4.4)	4.5 (2.7, 6.3)	

Table 2 presents the final characteristics of the study population by treatment group. It is important to emphasize that statistically significant differences were found between intervention and control groups with regard to knowledge about eating and physical activity, as well as between the ratings for both variables and self-efficacy.

**Table 2:** Final Characteristics of study sample, by treatment group

		Intervention	Control	
		n = 498	n = 499	*p*
**Sex (%)**				
	Females	52.1 (47.7,56.5)	49.6 (45.2,54.0)	0.43
	Males	47.9 (43.5, 52.3)	50.4 (46.0,54.8)	
**Age (y) (%)**				
	< 10	0.5 (0.0,1.3)	0.6 (0.0,1.2)	0.82
	10	52.1 (47.7, 56.5)	53.0 (48.6,57.4)	
	11	41.1 (36.8,45.4)	39.0 (34.7,43.3)	
	12	4.6 (2.8,6.4)	4.8 (2.9,6.7)	
	≥ 13	1.6 (0.5,2.7)	2.6 (1.2,4.0)	
**Socioeconomic Index (SEI) (%)**				
	Low	34.7 (30.6,38.9)	34.4 (30.2,38.6)	0.12
	Medium	33.9 (29.8,38.1)	39.4 (35.1,43.7)	
	High	31.3 (27.3,35.4)	26.2 (22.3,30.1)	
**Overweight**				
	Females	21.8 (16.8,26.9)	22.8 (17.5,28.0)	0.32
	Males	16.3 (11.6,21.0)	20.3 (15.3,25.3)	
**Obesity (%)**				
	Females	8.8(5.4,12.3)	9.8 (6.1,13.5)	0.98
	Males	16.3 (11.6,21.0)	16.3 (11.7,20.9)	
**Body Mass Index (BMI) (X)**				
	Females	45.3 (31.8,58.9)	43.5 (29.3,57.5)	0.85
	Males	54.3 (38.8,69.8)	60.8 (45.4,76.2)	0.56
**Dietary intake (X)**				
	Energy (Kcal)	1829.0 (1761.3,1896.6)	1818.7 (1743.4,1894.0)	0.84
	Carbohydrates (g)	337.0 (323.4,350.6)	341.0 (325.7,356.2)	0.71
	Lipids (g)	37.9 (36.3,39.6)	35.9 (34.3,37.5)	0.08
**Physical Activity (%)**				
	Non active	13.8 (10.8,16.8)	16.3 (13.0,19.5)	0.53
	Moderately active	13.4 (10.4,16.4)	13.5 (10.5,16.5)	
	Active	72.9 (69.0,76.8)	70.3 (66.3,74.3)	
**Knowledge (X)**				
	Eating	8.2 (8.1,8.4)	7.7 (7.6,7.8)	0.000
	Physical Activity	7.8 (7.7,7.9)	7.6 (7.5,7.7)	0.028
*Eating (%)*				
	passed > 6	91.0 (88.5,93.5)	86.1 (83.1,89.2)	0.016
	failed < 6	9.0 (6.5,11.5)	13.9 (10.8,16.9)	
*Physical Activity*				
	passed > 6	92.2 (89.8,94.5)	91.8 (89.3,94.2)	0.81
	failed < 6	7.8 (5.5,10.2)	8.2 (5.8,10.7)	
**Self-efficacy (X)**				
	Eting	9.0 (8.9,9.1)	8.9 (8.8,9.0)	0.044
	Physical Activity	7.1 (7.0,7.3)	6.9 (6.7,7.0)	0.014
*Eating*				
	High	98.2 (97.0,99.4)	96.0 (94.3,97.7)	0.038
	Medium	1.8 (0.6,3.0)	4.0 (2.3,5.7)	
	Low	**-**	-	
*Physical Activity*				
	High	53.1 (48.7,57.9)	44.8 (40.4,49.2)	0.028
	Medium	43.7 (39.3,48.0)	52.0 (47.6,56.4)	
	Low	3.2 (1.7,4.8)	3.2 (1.7,4.8)	

### Effects on shift in body mass index categories

Table 3 shows the effects of the strategy on the change in or maintenance of BMI by type of treatment (IG and CG) and by stage (baseline and final), adjusting for sex, age, socioeconomic status, physical activity, consumption of carbohydrates, fats, perception of children regarding food and physical activity (auto-efficacy) and knowledge.

**Table 3:** Logistic Ordinal Model to observe the probability of change in BMI from Normal to Overweight and Overweight to Obesity, by stage and intervention period

BMI	Odds Ratio (OR)	*p*	95% Confidence Interval	
**Overweight**				
Treatment (TR) (control)	1.00			
Treatment (TR) (intervention)	0.45	0.243	0.12	1.71
Stage (baseline)	1.00			
Stage (final)	0.93	0.413	0.77	1.11
Interaction TR*stage	0.89	0.225	0.73	1.11
Sex(female)	0.8	0.135	0.6	1.1
Age	0.82	0.513	0.78	1.13
SEI (Low)	1.00			
SEI(Medium)	1.51	0.011	1.10	2.07
SEI(High)	1.82	< 0.001	1.30	2.56
Carbohydrates	0.99	0.259	0.99	1.00
Lipids	1.05	0.229	1.01	1.01
PA (moderate actives)	1.01	0.573	0.79	1.52
PA (actives)	1.03	0.843	0.79	1.32
Eating notes	1.02	0.551	0.94	1.11
PA notes	0.93	0.209	0.82	1.04
PA auto-efficacy notes	1.15	< 0.001	1.07	1.24
Eating auto-efficacy notes	0.92	0.099	0.83	1.02
Interaction TR* Carbohydrates	0.99	0.060	0.99	1.00
Interaction TR* PA notes	1.16	0.090	0.98	1.37
**Obesity**				
Treatment (TR) (control)	1.00			
Treatment (TR) (intervention)	0.34	0.251	0.06	2.13
Stage(baseline)	1.00			
Stage(final)	1.29	0.065	0.98	1.70
Interaction TR*stage	0.68	0.010	0.51	0.91
Sex(female)	0.56	0.002	0.39	0.80
Age	0.89	0.399	0.68	1.17
SEI (Low)	1.00			
SEI (Medium)	168	0.041	1.02	2.75
SEI(High)	2.99	< 0.001	1.83	4.91
Carbohydrates	1.00	0.705	0.99	1.00
Lipids	0.99	0.270	0.98	1.00
PA (moderate actives)	0.83	0.436	0.51	1.34
PA (actives)	1.06	0.747	0.73	1.55
Eating notes	1.07	0.237	0.95	1.21
PA notes	0.87	0.146	0.73	1.05
PA auto-efficacy notes	1.12	0.042	1.00	1.24
Eating auto-efficacy notes	0.90	0.130	0.79	1.03
Interaction TR* Carbohydrates	0.99	0.200	0.99	1.00
Interaction TR* PA notes	1.29	0.044	1.01	1.59

### Effects on shifting from normal to overweight

**Figure 2:**
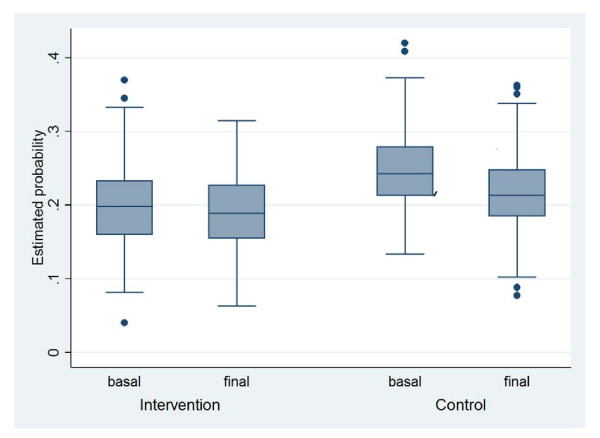
**Probability of Change in BMI from Normal to Overweight**.

Figure 2 shows the probability of shifting from a normal BMI to overweight among school children by stage of intervention and type of treatment. The probability of going from normal to overweight between baseline and the final intervention stage is not significant. For the IG, the estimated probability is 21.1% (95%CI 19.8, 22.1%) at baseline and 20% at the final stage (95%CI 18.4, 21.4).

For the CG, the estimated probability of moving from a normal BMI to overweight was 25.7% (CI 95% 24.1, 27.0) at baseline and 22.5% (95%CI 20.9, 23.7) during the final stage. No significant differences were found between intervention groups or study stages (Figure 2).

When adjusting for SEI, the probability of overweight shows a statistically significant risk for medium and high SEI as compared to low SEI (OR = 1.51; 95%CI 1.1, 2.1 and OR = 1.82; 95%CI 1.3, 2.6, respectively).

Regarding perceived self-efficacy, it was found that with higher food self-efficacy the risk of overweight (OR = 0.92 95%CI 0.83, 1.0) is reduced. However, in relation to self-efficacy in physical activity, an increased risk of overweight was found to be associated with increased self-efficacy (OR = 1.51 95%CI 1.1, 2.1).

In addition, the effect of carbohydrate consumption by type of intervention showed a lower risk of overweight associated with the combined interaction effect of the intervention and the consumption of carbohydrates (OR = 0.99 95%CI 0.99, 1.0).

### Effect on shifting from overweight to obesity

**Figure 3:**
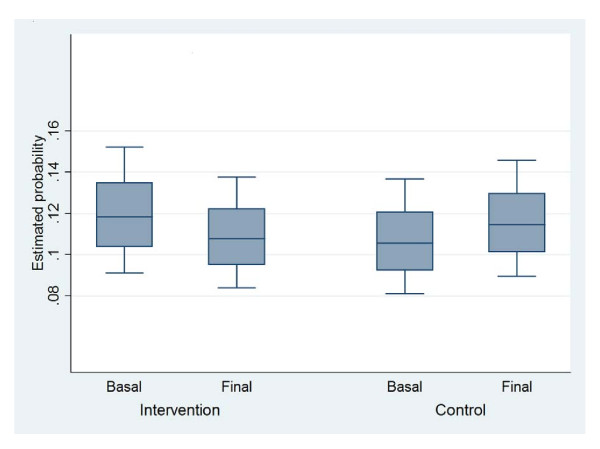
**Probability of Change in BMI from Overweight to Obesity**.

The probability of shifting from overweight to obesity showed a slight increase (*p *= 0.065); the estimated probability (EP) of obesity between baseline and the final stage for the IG decreased 1% (initial EP = 11.8%, 95%CI 9.0, 15.2; final EP = 10.8, 95%CI 8.4, 13.8) and for the CG it increased 0.9% (Initial EP = 10.6%, 95%CI 8.1, 13.7; final EP = 11.5, 95%CI 9.0, 14.6) (Figure 3). This is explained by the interaction between the intervention and the stage, which is the average odd time corrected treatment effect of the difference-in-differences, and is statistically significant (*p *= 0.01) (OR = 0.68, 95%CI 0.52, 091).(Figure 3).

In addition, being a girl showed a protective effect (OR = 0.56, 95%CI 0.39, 0.80), and higher socioeconomic status increased children's probability of moving from overweight to obese in relation to children from the lowest tertile. There is a significant risk at the medium SEI as compared to the lowest (OR = 1.68, 95%CI 1.02, 2.75), and an even higher risk for children from a high SEI in relation to the lowest (OR = 3.00, 95%CI 1.83, 4.91).

In terms of self-efficacy, children who perceived a higher risk regarding physical activity had an increased risk of shifting from overweight to obesity (OR = 1.12, 95%CI 1.00, 1.24), and an increased risk of obesity was associated with the combined effect of physical activity skills and the intervention (OR = 1.20, CI 95% 1.01, 1.59).

## Discussion and Conclusions

The main finding of the present study is that the strategy "Nutrition on the Go" had a small but statistically significant effect on reducing the probability of shifting from the overweight to the obesity category after 6 months of intervention. This study also documented a decreasing effect on the shift from the normal to the overweight categories during 6 months of intervention.

Several studies have shown similar results, in which the implementation of strategies that combine proper nutrition, promotion of consumption of fruits and vegetables, the consumption of pure water and performing physical activity in school areas for periods ranging from 4 to 12 months help to maintain or slightly improve BMI. For example, in meta-analysis studies conducted in 2008 [13] -- that analyzed studies of 22 interventions lasting less than 1 year, in which physical activity was increased and dietary changes were made in participants under 18 years old who were residents of Asia, South America, Europe and North America--authors reported that nearly all the studies showed some improvement in diet or physical activity, with a small but positive effect on BMI.

Another meta-analysis [14] showed similar findings, which included 38 controlled trials lasting at least 12 months, where all the studies showed significant and positive effects of the interventions on maintaining BMI values or categories, relative to the control groups. Likewise, another study [15] describes a compilation of 15 randomized controlled community trials and 4 non-randomized trials conducted in different countries, in which interventions among school age children had significant but modest effects on reducing body weight. All cases mention the need to standardize the design and implementation of strategies.

In addition, the study herein found a strong association between the probability of overweight and obesity in children and higher socioeconomic status. These findings have not been fully explained. It seems that in industrialized and developing countries patterns of obesity are related to the socioeconomic status of the family, with a trend toward greater risk of weight gain for urban and high-income children due to easy access to diets rich in energy and poor in nutrients, as well as additional variables such as access to transportation and lack of access to healthier alternatives [47]. On other hand, greater risk of obesity has been reported among children in Western societies where disadvantaged socioeconomic environments prevail than those among the wealthiest groups. Though the reasons for this are unclear, there are dietary differences that may contribute to this phenomenon, such as a high consumption of energy-dense food, which is often less expensive [48].

The results of the present study are similar to those reported elsewhere [22], in which a literature review was conducted on the effectiveness of 50 programs (experimental and quasi-experimental) to reduce obesity in schools. These studies included interventions using the school curriculum or preventive environmental interventions, with a baseline assessment and at least 6 months of intervention, and reported outcomes in terms of overweight measurements. The authors concluded that interventions are most effective for females when they include social aspects and for males when they involve physical activity.

Moreover, self-efficacy and children's perception about their own ability to perform physical activity and healthy eating is now considered to be more related to changes in behavior related to obesity risk factors. It is thus important to identify elements that affect this among obese children.

With regard to the results found in this study about children's perception about self-efficacy in physical activity and the presence of more overweight and obesity, this can initially be explained by the evaluation instrument. Though validated for Mexican children [49], the authors of the same study note the instrument's limitations due to the level of cognitive development and the degree of understanding shown by their participants. Therefore, they had to use a dichotomous scale (yes or no) rather than the 5-point scales commonly used for this kind of instrument.

Such a restriction in the variability of the responses could also create difficulties for detecting the relationship between self-efficacy and physical activity [43].

Moreover, there are factors such as those reported in Chile, where overweight or obese children believe to have the capacity to perform physical activity, but show limited ability to perform it when compared to children with normal weight, due to being less fit and fear of falling and/or causing injury to themselves [50]. This is contrary to that reported in another study [51], showing that obese students had significantly less self-efficacy and their parents were less active.

Likewise, the present study finds that the greater the knowledge about physical activity, the greater the probability of obesity. In this regard, a study in Chile [29] --whose objective was to evaluate the effectiveness of a school program for the prevention of childhood obesity in children--showed that one of the main factors that negatively influence the implementation of the intervention was the lack of teacher participation in activities such as teaching about healthy eating and physical activity to children in the classroom.

The design of the present study and randomization of schools enabled reducing the ascertainment bias of the study population to a minimum. Thus, the results attributed to the strategy reflect its effectiveness in maintaining the BMI of school children and decreasing the probability of obesity.

One of the limitations of the present study is that it was conducted only in the school environment, where children are present for 4 1/2 hours, so larger effects cannot be expected since children spend the rest of the day in obesigenic environments. It is logical to expect changes in the behavioral variables. Another limitation is the duration of the intervention, which lasted only 6 months; a longer intervention is needed in order to observe the impacts. Nevertheless, the literature review shows that intervention lengths for some studies ranged from 9 weeks to 3 years and that intervention of all durations successfully reduced obesity among overweight or obese children. There is also some evidence that suggests that shorter treatment periods may be associated with larger treatment effects [52,53].

Another aspect that is important to consider is that the forms (intensity and duration) of physical activity of intervention programs could have an effect on children's BMI. In addition, the duration and frequency of these activities is much too short to have any serious effect on children's BMI. There were probably also many factors that were not controlled but that could have influenced children's BMI over the 6-month intervention period.

An important aspect to consider in this type of intervention in schools in Mexico is little or no participation of teachers and principals, which may be a factor that limits the success of the strategy. In this regard, we provided motivation and awareness strategies targeted at teaching personnel to make them aware that obesity can be a problem with serious repercussions for their own health. Furthermore, because of the suspension of school classes for various reasons-assessments, holidays and festivals, among others--it is crucial to offer educational activities to prevent overweight and obesity in schools as part of the school curriculum in order to ensure their ongoing continuation in the school environment.

In this regard, it would be advisable to encourage healthy habits among children at earlier stages in order to create healthy students; the present study involved children at a stage during which certain habits are difficult to change.

In conclusion, the "Nutrition on the Go" strategy is considered to be effective for maintaining the BMI of school children. The authors suggest that policies for interventions to prevent obesity in schools in Mexico should consider changes within the school environment itself through actions such as improving physical education classes and creating more aggressive nutrition policies. For greater impact, the inclusion of parents and teachers is recommended, as well as government officials, communities and civil society as elements in healthy lifestyles as well as the development and welfare of society. The authors are currently beginning the work with parents and teachers to engage them in the strategy, and believe that they are fundamental actors in changing the behavior of children.

## Endnote

^a^Corresponds to the calculation of the difference in grams per year of age for individuals of normal height between 0 and 19 years of age. This represents 0.45 BMI units. This increase in grams reflects the observable differences in the sample

## Competing interests

The authors declare that they have no competing interests.

## Authors' contributions

The authors' responsibilities were as follows TSH: coordinated the data analysis and interpretation and wrote the manuscript; CM, AJ and IM: helped with the interpretation and contributed to the writing of the manuscript; TSH, CM and AJ: contributed to the conception and design of the study; CA and AS: coordinated the data collection phase; IM: conducted the data analysis and provided statistical expertise. All authors read and approved the final manuscript.

## Author disclosures

T. Shamah Levy, C. Morales Ruán, C. Amaya Castellanos, A. Salazar Coronel, A. Jiménez Aguilar, Ignacio Méndez Gómez Humarán.

## Pre-publication history

The pre-publication history for this paper can be accessed here:

http://www.biomedcentral.com/1471-2458/12/152/prepub
